# Japanese Lifestyle during Childhood Prevents the Future Development of Obesity among Japanese-Americans

**DOI:** 10.1371/journal.pone.0120804

**Published:** 2015-03-25

**Authors:** Mami Shiwa, Masayasu Yoneda, Shuhei Nakanishi, Kenji Oki, Kiminori Yamane, Nobuoki Kohno

**Affiliations:** Department of Molecular and Internal Medicine, Graduate School of Biomedical & Health Sciences, Hiroshima University, Hiroshima, Japan; The University of Tokyo, JAPAN

## Abstract

**Objective:**

To evaluate whether a Japanese lifestyle during childhood could protect against the future development of obesity-associated metabolic diseases by comparing native Japanese with Japanese-Americans in whom genetic factors are the same.

**Methods:**

Study subjects were 516 native Japanese and 781 Japanese-Americans who underwent medical examinations between 2007 and 2010. Japanese-Americans were divided into 444 first-generation immigrants (JA-1), who were born in Japan, and 337 second- or later-generation descendants (JA-2), who were born in the United States. The JA-2 group was then divided into the *kibei* subgroup (N = 79), who had moved to Japan before the age of 18 years and later returned to the United States, and the non-*kibei* subgroup (N = 258), who had never lived in Japan.

**Results:**

The JA-2 group had the highest percentages of obesity, metabolic syndrome, and type 2 diabetes compared with native Japanese and JA-1. Furthermore, among JA-2, the prevalence of obesity and metabolic syndrome in the *kibei* subgroup was significantly lower than that in the non-*kibei* subgroup. The prevalence of diabetes in the kibei subgroup also tended to be lower than in the non-*kibei* subgroup.

**Conclusions:**

The prevalence of obesity and metabolic diseases differed with residence in Japan during childhood among Japanese-Americans. These findings indicate the possibility that Japanese lifestyle during childhood could reduce the future risks for obesity-associated metabolic diseases.

## Introduction

The immigration of Japanese people to the Hawaiian Islands began during the latter half of the 19th century. The lifestyle, including diet and amount of exercise, differed greatly between Japan and the United States at that time. Upon arrival in the United States, the lifestyles of many such Japanese immigrants underwent rapid westernization. Because Japanese-Americans, while genetically equivalent to native Japanese, have a considerably more westernized lifestyle, they are an appropriate population for investigating the effects of a westernized lifestyle on the incidence of metabolic diseases among Japanese individuals.

In 1970, we began to conduct a medical survey of Japanese-Americans in an epidemiological study called “the Hawaii—Los Angeles—Hiroshima Study” [1]. Since then, these surveys have been conducted every few years in Hawaii or Los Angeles, now totaling 23 times, and by the end of 2014 had included over 12,700 participants. We compared Japanese who reside in Japan and follow a Japanese lifestyle with Japanese-Americans who reside in the United States and follow a westernized lifestyle. Level of obesity was significantly greater in Japanese-Americans than in Japanese living in Hiroshima [2], as was the prevalence of type 2 diabetes [3, 4] and metabolic syndrome (MS) [5]. Furthermore, Japanese-Americans showed more rapidly progressing atherosclerosis than did native Japanese [6].

Few studies have described whether childhood lifestyle has a strong effect on the future development of obesity and diabetes mellitus [7–9]. In this study, Japanese-Americans were divided into two groups: those who were born and grew up in Japan and later emigrated to the United States (termed “first-generation Japanese-Americans”), and those who were born and grew up in the United States (termed “second- or later-generation Japanese-Americans”). Moreover, the second- or later-generation Japanese-Americans can be further subdivided into individuals who were born in the United States and grew up there, and individuals who were born in the United States but had an experience that living and being educated in Japan during their childhood and adolescence and then returning to the United States (termed the “*kibei* subgroup” [“*kibei*” is the Japanese term for “return to the United States”]). We hypothesized that the incidence of lifestyle-related diseases in people growing up with a Japanese lifestyle would be lower than in people growing up with a westernized lifestyle by generation or having *kibei* experience among Japanese-Americans.

The objective of this study was to determine whether a Japanese lifestyle during childhood would act as a buffer against the development of obesity and metabolic diseases. We compared the prevalence of obesity and metabolic diseases among four groups: native Japanese, first-generation Japanese-Americans, and second- or later-generation Japanese-Americans divided by *kibei* experience (*kibei* subgroup: resided in Japan during childhood and returned to the United States; non-*kibei* subgroup: grew up in the United States).

## Methods

### Subjects

Japanese and Japanese-Americans whose parents were both Japanese had participated in the medical survey, excluding potential participants below the age of 30 years or with a mixed ethnic background. The subjects of this study were 221 Japanese-Americans (132 men and 89 women) living in Hilo and Kona, on the Island of Hawaii in 2007; 560 Japanese-Americans (237 men and 323 women) living in Los Angeles, California in 2010; and 516 native Japanese (171 men and 345 women) living in Hiroshima in 2009. Japanese-Americans comprised 56.9% first-generation individuals, 18.1% second-generation, 22.1% third-generation, 2.8% fourth-generation, and 0.1% fifth-generation. We combined the second-, third-, fourth-, and fifth-generation into “second- or later- generations”. The first-generation Japanese-Americans and the second- or later-generation Japanese-Americans were abbreviated as “JA-1” and “JA-2”, respectively, in this study. Within JA-2, Japanese-Americans who moved to Japan before the age of 18 and stayed for at least five years were classified as the *kibei* subgroup, and individuals who had never lived in Japan were defined as the non-*kibei* subgroup. JA-2 consisted of 79 *kibei* (42 men and 37 women) and 258 non-*kibei* (153 men and 105 women). JA-2 who had lived in Japan during childhood for less than 5 years or moved to Japan after the age of 19 years were excluded from the analysis.

Due to a significant difference in average age between native Japanese, JA-1, and JA-2, study participants were separated into three age groups: 30–49 years, 50–69 years, and ≥ 70 years old. When we compared the *kibei* subgroup with the non-*kibei* subgroup, due to the small number of JA-2 participants in the 30–49 age group, JA-2 was separated into two age groups incorporating the 50–69 category: < 70 years old and ≥ 70 years old.

All subjects provided their written informed consent to participate in the examinations. This study was approved by the ethics committee of Hiroshima University and the Councils of the Hiroshima Kenjin-Kai Association in Hawaii and Los Angeles.

### Measurements

Medical examinations took place in the morning after an overnight fast. Each subject completed an interview, physical and blood pressure (BP) measurements, and venous blood sampling. Waist circumference was measured at the level of the umbilicus. Body mass index (BMI) was calculated by dividing weight (kg) by height squared (m^2^). An oral glucose tolerance test (OGTT) was performed on subjects without diabetes. Each blood sample was centrifuged, and the obtained serum samples were immediately frozen and stored until analysis. Serum glucose levels were measured by the hexokinase method; insulin levels by double-antibody radioimmunoassay; total cholesterol and triglyceride levels by an enzymatic method; and high-density lipoprotein (HDL) cholesterol levels by homogenous assay.

### Diagnostic criteria

The definitions for obesity, MS, and diabetes were as follows. Obesity was defined as BMI ≥ 25 kg/m^2^ [10].

MS was diagnosed according to International Diabetes Federation criteria [11]. These criteria include an essential component of central obesity (a waist circumference ≥ 90 cm in men and ≥ 80 cm in women). Individuals require two or more of the following four factors in addition to central obesity. The first component is raised BP (systolic BP ≥ 130 mmHg and/or diastolic BP ≥ 85 mmHg) or treatment of previously diagnosed hypertension. The second component is raised triglycerides level (≥ 150 mg/dl) or specific treatment for this lipid abnormality. The third component is reduced HDL cholesterol level (< 40 mg/dl in men and < 50 mg/dl in women), or specific treatment for this lipid abnormality. The final component is raised fasting glucose level (≥ 100 mg/dl) or treatment of previously diagnosed type 2 diabetes.

Diabetes was diagnosed as fasting glucose level ≥ 126 mg/dl and/or two-hour post-load (2-h) glucose level ≥ 200 mg/dl after an OGTT [12], or as treatment of previously diagnosed diabetes.

### Statistical analysis

Values are expressed as numbers, means ± SD, medians (25th–75th percentile levels), or percentages. Fasting glucose, 2-h glucose, fasting insulin, homeostasis model assessment-insulin resistance (HOMA-IR) [13], and triglycerides were analyzed after logarithmic transformation, because distributions of these parameters were skewed. Mean differences among the groups were tested by a multiple regression analysis or an ANCOVA, adjusting for age and sex, followed by the Bonferroni multiple comparison test. *χ* ^2^ test was used to evaluate frequency differences among the groups. A *P*-value of < 0.05 was considered statistically significant. Statistical analyses were performed with the software packages SPSS version 19.0 (IBM Corp., Armonk, NY) and SAS version 8.2 (SAS Institute, Cary, NC).

## Results

### Comparison of native Japanese and Japanese-Americans by generation

Table 1 shows the demographic and medical characteristics of this study’s subjects. For JA-1, the average age at immigration to the United States was 27.0 ± 8.4 years old, with the mean duration of stay in the United States being 35.5 ± 14.1 years. BMI was significantly higher in JA-2 than in both native Japanese and JA-1. Waist circumference was significantly higher in native Japanese and JA-2 than in JA-1. Among men, waist circumference did not differ significantly among the three groups. On the other hand, among women, waist circumference was significantly higher in native Japanese and JA-2 than in JA-1. Systolic BP was significantly higher in JA-1 than in native Japanese. Diastolic BP was significantly higher in JA-1 than in native Japanese and JA-2. No significant difference was observed in fasting glucose level and 2-h glucose level, but fasting insulin level and HOMA-IR were significantly higher in JA-1 than in native Japanese. Furthermore, those values were significantly higher in JA-2 than in JA-1. Total cholesterol level was significantly lower in JA-2 than in native Japanese and JA-1. Triglycerides were significantly higher in both JA-1 and JA-2 than in native Japanese. Compared with native Japanese and JA-1, JA-2 had a significantly higher rate of treatment for hypertension, dyslipidemia, and diabetes.

**Table 1:** Characteristics of study subjects.

	Japanese	Japanese-Americans	
		JA-1	JA-2
N (Men/Women)	516 (171/345)	444 (174/270)	337 (195/142)
Age, years	60.4 ± 12.8	62.4 ± 11.8^a^	68.0 ± 14.2^b^ ^c^
BMI, kg/m^2^	23.0 ± 3.2	23.3 ± 3.1	24.8 ± 4.2^b^ ^c^
Waist circumference, cm	83.6 ± 9.1	79.8 ± 10.3^a^	86.1 ± 11.8^c^
Men, cm	86.2 ± 7.8	85.2 ± 8.7	87.6 ± 11.2
Women, cm	82.3 ± 9.5	76.4 ± 9.7^a^	84.0 ± 12.4^c^
Systolic BP, mmHg	127.0 ± 18.1	133.3 ± 18.9^a^	134.8 ± 17.7
Diastolic BP, mmHg	77.8 ± 11.0	82.7 ± 11.0^a^	77.1 ± 10.4^c^
Fasting glucose, mg/dl	93.0 (88.0–99.0)	91.0 (85.0–100.0)	92.0 (86.0–101.0)
2-h glucose, mg/dl	119.0 (102.0–144.0)	113.0 (94.0–144.0)	125.0 (99.0–157.0)
Fasting insulin, μU/ml	4.0 (3.2–5.9)	5.1 (3.5–10.0)^a^	6.1 (3.7–13.5)^b^ ^c^
HOMA-IR	0.9 (0.7–1.5)	1.2 (0.8–2.3)^a^	1.5 (0.8–3.3)^b^ ^c^
Total cholesterol, mg/dl	209.3 ± 33.0	210.0 ± 41.8	198.7 ± 38.9^b^ ^c^
HDL cholesterol, mg/dl	62.5 ± 14.4	61.3 ± 16.5	63.8 ± 16.7^b^ ^c^
Triglycerides, mg/dl	91.0 (66.0–125.0)	115.0 (80.0–168.8)^a^	110.0 (77.0–166.0)^b^
Treatment for			
hypertension, %	20.2	31.3^a^	43.6^b^ ^c^
dyslipidemia, %	21.3	22.3	37.4^b^ ^c^
type 2 diabetes, %	8.3	9.5	15.4^b^ ^c^
Current smoking, %	11.4	14.4	8.0^c^
Alcohol drinking, %	29.1	27.8	19.7^b^ ^c^

JA-1, first-generation Japanese-Americans; JA-2, second- or later-generation Japanese-Americans; BMI, body mass index; BP, blood pressure; 2-h, two-hour post-load; HOMA-IR, homeostasis model assessment-insulin resistance; HDL, high-density lipoprotein.

Values are expressed as numbers, means ± SD, medians (25th–75th percentile levels), or percentages.

The parameters were analyzed after adjusting for age and sex.

^a^ *P* < 0.05 between Japanese and JA-1.

^b^ *P* < 0.05 between Japanese and JA-2.

^c^ *P* < 0.05 between JA-1 and JA-2.

The prevalence of obesity, MS, and type 2 diabetes among native Japanese and Japanese-Americans by generation is shown in Table 2. There was a significant trend of increasing obesity rates among native Japanese, JA-1, and JA-2, in that order. We observed a similar trend among both men and women and among all age groups. There was a significant trend of increasing prevalence of MS among native Japanese, JA-1, and JA-2. We observed a similar trend among men and women. There was a significant trend among the age groups of 30–49 years and ≥ 70 years old. The prevalence of type 2 diabetes was 12.2%, 18.9%, and 22.0% for native Japanese, JA-1, and JA-2, respectively. There was a significant trend among all subjects, both men and women. We observed a significant trend among the age group of ≥ 70 years old.

**Table 2:** Prevalence of obesity, metabolic syndrome, and type 2 diabetes among native Japanese and Japanese-Americans by generation

	Obesity				Metabolic syndrome				Type 2 diabetes			
	Japanese	JA-1	JA-2	*P* for trend	Japanese	JA-1	JA-2	*P* for trend	Japanese	JA-1	JA-2	*P* for trend
	(N = 516)	(N = 444)	(N = 337)		(N = 516)	(N = 444)	(N = 337)		(N = 516)	(N = 444)	(N = 337)	
All, %	25.2	28.6	43.6	< 0.001	24.0	22.3	38.0	< 0.001	12.2	18.9	22.0	<0.001
Men, %	30.4	39.7	44.1	0.008	19.9	20.1	32.8	0.003	14.0	24.7	24.6	0.016
Women, %	22.6	21.5	43.0	< 0.001	26.1	23.7	45.1	0.001	11.3	15.2	18.3	0.033
30–49 years, %	25.7	21.7	48.8	0.019	11.9	11.7	30.2	0.014	5.5	3.3	14.0	0.127
50–69 years, %	26.9	30.6	51.8	< 0.001	28.8	22.4	42.1	0.077	13.7	18.8	20.2	0.073
≥ 70 years, %	21.3	27.9	37.2	0.002	24.3	27.1	37.2	0.011	14.7	26.4	25.0	0.039

JA-1, first-generation Japanese-Americans; JA-2, second- or later-generation Japanese-Americans.

### Comparison of the *kibei* and non-*kibei* subgroups

The characteristics of the JA-2 generations by *kibei* status are shown in Table 3. For the *kibei* subgroup, the average age at arrival in Japan was 4.3 ± 4.1 years old and that at departure from Japan was 18.8 ± 5.3 years, with the mean duration of stay in Japan being 14.9 ± 5.8 years (minimum duration of 5 years and maximum duration of 33 years). The *kibei* subgroup was significantly older and had higher diastolic BP than the non-*kibei* subgroup. Although BMI in the non-*kibei* was higher, but not significantly so, than in the *kibei* subgroup, the non-*kibei* had significantly higher waist circumference than the *kibei* subgroup. There were no significant differences between subgroups on fasting insulin level and HOMA-IR. Rate of treatment for hypertension, dyslipidemia, and diabetes were not significantly different.

**Table 3:** Characteristics of the JA-2 generations by *kibei* status.

	*Kibei*	Non-*kibei*
N (Men/Women)	79 (42/37)	258 (153/105)
Age, years	72.1 ± 11.0	66.8± 14.9^a^
BMI, kg/m^2^	23.9 ± 3.7	25.1 ± 4.3
Waist circumference, cm	82.7 ± 11.5	87.1 ± 11.7^a^
Men	85.3 ± 9.9	88.2 ± 11.5
Women	79.9 ± 12.7	85.5 ± 11.9
Systolic BP, mmHg	133.6 ± 17.3	135.3 ± 17.8
Diastolic BP, mmHg	80.6 ± 10.5	76.0 ± 10.1^a^
Fasting glucose, mg/dl	91.0 (84.0–98.0)	93.0 (86.0–103.0)^a^
2-h glucose, mg/dl	127.5 (97.0–164.3)	124.5 (99.5–153.8)
Fasting insulin, μU/ml	6.2 (3.7–13.8)	6.0 (3.7–13.5)
HOMA-IR	1.3 (0.8–3.0)	1.5 (0.8–3.4)
Total cholesterol, mg/dl	197.7 ± 42.2	199.0 ± 37.9
HDL cholesterol, mg/dl	60.4 ± 16.0	64.8 ± 16.8^a^
Triglycerides, mg/dl	106.0 (73.0–165.0)	112.5 (78.0–166.0)
Treatment for		
hypertension, %	46.8	42.6
dyslipidemia, %	35.4	38.0
type 2 diabetes, %	15.2	15.5
Current smoking, %	24.4	18.3
Alcohol drinking, %	10.1	7.4

BMI, body mass index; BP, blood pressure; 2-h, two-hour post-load; HOMA-IR,

homeostasis model assessment-insulin resistance; HDL, high-density lipoprotein.

Values are expressed as numbers, means ± SD, medians (25th–75th percentile levels), or percentages.

The parameters were analyzed after adjusting for age and sex.

^a^ *P* < 0.05 between *kibei* and non-*kibei*.

The prevalence of obesity, MS, and type 2 diabetes among the JA-2 generation by *kibei* status is shown in Table 4. We found the prevalence of obesity and MS in the non-*kibei* to be significantly higher than that in the *kibei* subgroup. The prevalence of type 2 diabetes in the non-*kibei* was higher, but not significantly so, than that in the *kibei* subgroup. According to gender, the rates of obesity, MS, or diabetes in the non-*kibei* were higher than in the *kibei* subgroup both in men and women but not significantly so. There were no significant differences in the rates of obesity, MS, or diabetes between groups under the age of 70. For those over 70 years, however, the rates of obesity and MS in the non-*kibei* were significantly higher than in the *kibei* subgroup, with the same (non-significant) tendency for type 2 diabetes.

**Table 4:** Prevalence of obesity, metabolic syndrome, and type 2 diabetes among the JA-2 generations by *kibei* status.

	Obesity			Metabolic syndrome			Type 2 diabetes		
	*Kibei*	Non-*kibei*	*P*-value	*Kibei*	Non-*kibei*	*P*-value	*Kibei*	Non-*kibei*	*P*-value
	(N = 79)	(N = 258)		(N = 79)	(N = 258)		(N = 79)	(N = 258)	
All, %	31.6	47.3	0.014	26.6	41.5	0.017	19.0	22.9	0.466
Men, %	33.3	47.1	0.113	21.4	35.9	0.076	21.4	25.5	0.588
Women, %	29.7	47.6	0.059	32.4	49.5	0.072	19.0	16.2	0.702
< 70 years, %	48.3	51.6	0.749	34.5	39.8	0.593	24.1	17.2	0.384
≥ 70 years, %	22.0	43.9	0.009	22.0	43.1	0.009	16.0	28.5	0.084

## Discussion

The present study is unique in revealing that temporary residence in Japan during childhood had a protective effect against the development of obesity and metabolic diseases, despite the increased prevalence of obesity in Japanese-Americans due to living a westernized lifestyle. Native Japanese, first-generation Japanese-Americans, and second- or later-generation Japanese-Americans (individuals in both the *kibei* and non-*kibei* groups) were separated by birthplace, growing up place, and living place (between Japan and the United States). Intriguingly, they had significantly different prevalence rates of obesity and metabolic diseases, which were caused by their different lifestyles in childhood.

In this 2007 to 2010 study, as in our previous reports, Japanese-Americans had a significantly higher prevalence of obesity, MS, and type 2 diabetes than did native Japanese [2–5]. Furthermore, we found that the longer Japanese-Americans had been exposed to a westernized lifestyle, the more likely they were to develop obesity and metabolic diseases by generational comparison of native Japanese and Japanese-Americans. Place of birth and the length of time spent in the United States among Asian-American immigrants were correlated with increased risks of obesity and diabetes [14, 15], which was consistent with our earlier report. We previously reported that the consumption of animal protein, animal fat, and simple carbohydrates increased across groups in order of native Japanese, JA-1, and JA-2, in spite of equal total energy intake among the three groups in the 1990s [16]. It is conceivable that the generational differences of prevalence of obesity and lifestyle-related diseases were significantly attributable to dietary acculturation across generations among Japanese-Americans.

Another interesting result from our study is that the prevalence of obesity and MS in the *kibei* subgroup was lower than that in the non-*kibei* subgroup, and more similar to that of JA-1, regardless of generation (as shown in Tables 2 and 4). Because childhood and adolescent lifestyle affected dietary patterns [17] and physical activity [18] even into adulthood, early lifestyle could be associated with the risks of the development of obesity and diabetes in later life [7–9]. Hankin et al. showed that Japanese-Americans who had lived in Japan during childhood (the so-called *kibei* subgroup) tended to routinely eat Japanese food compared with second-generation Japanese-Americans who had never lived in Japan (the so-called non-*kibei* subgroup) [19]. These reports suggested that the JA-1 generation and the *kibei* subgroup consumed a Japanese diet and were physically active during childhood in Japan, and maintained a Japanese lifestyle throughout their lifetime even after their return to the United States. As a result, they might be protected against developing obesity and MS in their later years.

On the other hand, there was no significant difference in the prevalence of type 2 diabetes between the *kibei* and the non-*kibei* subgroups. We previously reported that high HOMA-IR values were useful for predicting the development of type 2 diabetes among Japanese-Americans [20]. Visceral adiposity is also known as a strong risk factor predicting the development of glucose intolerance [21] and diabetes [22] in Japanese-Americans. We found that HOMA-IR in Japanese-Americans was higher than in native Japanese, but there was no significant difference between the *kibei* and the non-*kibei* subgroups. This result supports the idea that the incidence of diabetes in Japanese individuals, unlike that of obesity and MS, is mainly attributable to insulin resistance and aging rather than lifestyle during childhood [23].

This study has several limitations. The primary among them was the limited number of participants. We were not able to separate individuals into narrower age categories (per 10 years), and the gender distribution significantly differed among Japanese, JA-1, and JA-2. Moreover, due to the small number of young *kibei* subgroup participants, we combined them into the age group of < 70 years old. As a result, in the < 70 years age group, the *kibei* subgroup was significantly older than the non-*kibei* subgroup, and therefore the *kibei* subgroup might have had a conversely higher prevalence of type 2 diabetes than the non-*kibei* subgroup. Second, the questionnaire items were insufficient for clarifying the background characteristics of each group. We did not evaluate diet, physical activity, and socioeconomic status, which would affect the development of obesity and lifestyle-related diseases according to both of generation and *kibei* status. Finally, the background characteristics of JA-1 were rather varied. Many young Japanese-Americans who had recently migrated from Japan to the United States were included in JA-1, which may explain the lack of a difference in the prevalence of obesity and MS between native Japanese and JA-1.

## Conclusions

We demonstrated that Japanese lifestyle during childhood was associated with a lower likelihood of developing obesity and MS among Japanese-Americans. Our findings suggest the possible importance of a traditional Japanese lifestyle in preventing the incidence of obesity-associated metabolic diseases.
